# The Effect of Early Beta-Blockade with Esmolol on Therapy Intensity Level in Adults with Severe Traumatic Brain Injury

**DOI:** 10.1089/neur.2024.0055

**Published:** 2024-10-02

**Authors:** Thomas Baumer, George Higginbotham, Kati Hayes, Matt Thomas, for the EBB-TBI Investigators

**Affiliations:** ^1^ Specialist Registrar in Intensive Care Medicine, Southmead Hospital, North Bristol NHS Trust, Bristol, UK.; ^2^ Academic Foundation Doctor, Southmead Hospital, North Bristol NHS Trust, Bristol, UK.; ^3^ Research Nurse, Southmead Hospital, North Bristol NHS Trust, Bristol, UK.; ^4^ Consultant in Intensive Care Medicine, Southmead Hospital, North Bristol NHS Trust, Bristol, UK.

**Keywords:** beta-blockade, cerebral edema, esmolol, intensive care unit, intracranial pressure, traumatic brain injury

## Abstract

Following severe traumatic brain injury (TBI), elevated catecholamine levels are associated with worsened secondary brain injury and poorer clinical outcomes. The mechanisms are uncertain but may include cerebral ischemia and blood–brain barrier disruption, with consequent cerebral edema manifesting as intracranial hypertension. Early beta-blockade (EBB) may mitigate these detrimental hyperadrenergic effects. Therapy Intensity Level (TIL) is a validated score that quantifies intracranial pressure (ICP)-lowering interventions, with higher TIL being a surrogate for more severe intracranial hypertension. In this *post hoc* secondary analysis of a dose-finding study of EBB with esmolol in adults with TBI, we compared summary TIL (TIL24) and domain TIL between patients who received esmolol and those who did not. The primary outcome was TIL24 for each 24-h epoch of the esmolol intervention period of 96 h. Baseline characteristics were comparable in the esmolol (E) and non-esmolol (NE) groups. Mean TIL24 was similar in both groups up to 48 h but then diverged. The mean (standard deviation) TIL24 score between 48 and 72 h was 4.8 (1.5) in group E versus 6.6 (5.4) in group NE and at 72–96 h 4.5 (1.5) in group E versus 7.0 (4.0) in group NE. TIL domain scores were lower in group E for hyperosmolar therapy, targeted temperature management, and surgical management (cerebrospinal fluid drainage, evacuation, or decompressive craniectomy). The association between esmolol use after TBI and the reduction in ICP-directed interventions is consistent with an effect of beta-blockade on reduction of cerebral edema. Further research is necessary to determine causality and mechanism.

## Introduction

Following traumatic brain injury (TBI), high catecholamine levels are associated with worse functional and survival outcomes.^1–3^ Catecholamines may exacerbate secondary brain injury through two mechanisms. First, local vasoconstriction and higher cerebral metabolic demand predispose to cerebral ischemia and cytotoxic edema. Alternatively, or additionally, catecholamine-induced endothelial injury may increase blood–brain barrier permeability and vasogenic edema.^4–6^ The result is the development of generalized cerebral edema.

Consequently, there is longstanding interest in the use of beta-blockers for neuroprotection after severe TBI. As early as 1992, the Lund University Hospital, Sweden, advocated their use due to protective effects on intracranial pressure (ICP).^7^ Subsequently, evidence from multiple retrospective observational studies suggests that beta-blockade is associated with improved clinical outcomes, possibly due to a reduction in hyperadrenergic-mediated secondary brain injury and cerebral edema.^6,8,9^

Cerebral edema typically presents as raised ICP, necessitating urgent ICP-lowering interventions.^4–6^ “Therapy Intensity Level” (TIL) is a validated, reliable research tool for quantifying ICP-targeted medical and surgical interventions in patients with severe TBI.^10,11^ TIL provides a surrogate measure of the severity of cerebral edema as well as insight into the impact of clinical management that might not be apparent from ICP readings alone.

We are researching the effect of early beta-blockade with esmolol in adults with severe TBI (EBB-TBI).^12^ Esmolol is an ideal beta-blocker in patients with TBI, being easily titrated to effect, with a short half-life (9 min) and a good safety profile.^6,8,9,12^ There is evidence that short-term (24 h) fixed dose esmolol infusion is associated with a statistically significant reduction in ICP of 2.5 mmHg for patients with intracranial hypertension after severe TBI, though the mechanism is not known.^13^ Here, we report a *post hoc* analysis of a phase 2a dose-finding study, EBB-TBI (ISRCTN 11038397),^12^ presenting the effect of esmolol on TIL as a surrogate of cerebral edema for longer infusions (96 h) titrated to heart rate in adults after severe TBI.

## Materials and Methods

EBB-TBI recruited adult patients with severe TBI (defined as a Glasgow Coma Score of 8 or less after resuscitation or prior to intubation) and ICP monitoring admitted to the intensive care unit (ICU) of the Major Trauma Centre at Southmead Hospital, Bristol, between December 30, 2020, and February 15, 2022. Major exclusion criteria were a perceived devastating brain injury, life- or limb-threatening extracranial injury, or contraindication to beta-blockade with esmolol. As patients lacked capacity due to the severity of head injury, a deferred model of consent was used based on the need for early intervention.^12^

In this *post hoc* analysis, we measure TIL scores in two groups: the esmolol (E) patient group (the initial cohort recruited to the EBB-TBI study) and a “non-esmolol” (NE) comparator group. The NE patient group were those who met eligibility criteria but were not enrolled for logistical reasons (e.g., lack of availability of study team). The *post hoc* analysis was approved by the South Central–Hampshire A Research Ethics Committee (reference 20/SC/0219).

The (E) group received an open-label esmolol infusion (Brevibloc Premixed 10 mg/mL solution, Baxter Healthcare Ltd) within 2 h of confirmation of eligibility and within 24 h of injury. Esmolol was titrated to achieve a 15% reduction in heart rate from baseline (the mean heart rate in the 4 h prior to confirmation of eligibility). Esmolol was administered for 96 h unless a specific reason in the study protocol mandated the infusion to be stopped sooner.^12^

Esmolol was initiated at predefined dose levels based on doses tolerated in a previous study.^14^ Actual body weight was used with a maximum permitted dose of 200 mcg/kg/min. Six patients were initiated at 5 mcg/kg/min (titrated upward by 2.5 every 30 min); subsequently, three patients were initiated at 10 mcg/kg/min (increments of 5 mcg/kg/min), and following this, three patients were initiated at 16 mcg/kg/min (increments of 8 mcg/kg/min).

Apart from a heart rate target in the E group, all physiological goals including ICP (<22 mmHg) and cerebral perfusion pressure (CPP, 60–70 mmHg) were the same in both groups. All patients received usual care according to unit protocols based on Brain Trauma Foundation guidelines and as directed by the treating clinicians.^15^

TIL scores were calculated for both groups in 24-h epochs (TIL24). “Summary TIL” quantifies the intensity of ICP-related interventions out of a maximum of 38 across eight domains including patient positioning (/1), sedation/metabolic suppression and neuromuscular blockade (/8), cerebrospinal fluid (CSF) drainage (/3), fluid loading and vasopressor therapy (/2), hyperventilation (/4), hyperosmolar therapy (/6), treatment of fever and hypothermia (/5), and surgery for refractory ICP (/9). “Domain TIL” in each group can be compared for any domain, and when combined, a higher overall TIL24 score suggests a worse degree of cerebral edema.^10,11^

The primary outcome was the TIL24 score in each epoch. Timing of 24-h epochs was taken from initiation of esmolol or, in the usual care group, from the comparable time after ICU admission (based on a median of 13 h from ICU admission to esmolol being initiated in the E group). The baseline period for TIL calculation was taken as the time in ICU prior to this.

Secondary outcomes were TIL by domain, overall summary TIL (over 96 h), duration of mechanical ventilation, length of stay (LoS, ICU, and hospital), and mortality (ICU, hospital, and 6 months). Safety of esmolol was assessed by mean lowest heart rate and CPP in each epoch. Descriptive statistics were only used as this is not a hypothesis testing study.

## Results

A total of 24 patients were analyzed, 12 in the E group and 12 in the NE group. Baseline characteristics were similar between groups (Table 1).

**Table 1.** Baseline Characteristics and Outcomes

	Esmolol (*n* = 12)	Non-esmolol (*n* = 12)
Baseline characteristics		
Age (years, mean/SD)	39.9 (17.2)	35.9 (12.9)
Sex (M/F)	8/4	11/1
Isolated TBI (*n*/%)	8 (66.7)	5 (41.7)
Mechanism of injury (*n*/%)		
Road traffic collision	5 (41.7)	2 (16.7)
Fall <2 m	2 (16.7)	0 (0)
Fall >2 m	2 (16.7)	1 (8.3)
Sports injury	2 (16.7)	2 (16.7)
Assault	0 (0)	4 (33.3)
Other	1 (8.3)	3 (25.0)
Initial CT scan findings (*n*/%)		
Skull fractures	6 (50.0)	8 (66.7)
Traumatic subarachnoid hemorrhage	7 (58.3)	5 (41.7)
Subdural hematoma	4 (33.3)	9 (75.0)
Extradural hematoma	2 (16.7)	1 (8.3)
Parenchymal injury	7 (58.3)	6 (50.0)
Diffuse axonal injury	0 (0)	1 (8.3)
Preintubation GCS (mean/SD)	6.5 (3.2)	5.8 (1.2)
Injury to ICU time (hours, mean/SD)	6.0 (0.2)	5.3 (7.4)
Baseline highest ICP (mmHg, mean/SD)	16.4 (13.9)	17.9 (9.9)
Study period highest ICP (mmHg, mean/SD)	20.5 (13.3)	22.6 (10.2)
Baseline summary TIL (mean/SD)	8.8 (3.7)	8.0 (3.6)
APACHE-II (mean/SD)	10.5 (6.5)	9.6 (4.1)
IMPACT predicted 6-month mortality (%, mean/SD)	26.3 (21.3)	27.9 (24.2)
Primary outcome		
TIL24 0–24 (mean/SD)	7.5 (5.2)	7.1 (3.1)
TIL24 24–48 (mean/SD)	5.4 (1.9)	5.8 (3.7)
TIL24 48–72 (mean/SD)	4.8 (1.5)	6.6 (5.4)
TIL24 72–96 (mean/SD)	4.5 (1.5)	7.0 (4.0)
Secondary outcomes		
Summary TIL all epochs (mean/SD)	5.6 (3.1)	6.0 (0.2)
Lowest heart rate (bpm, mean/SD)	53.5 (14.8)	58.9 (10.0)
Lowest cerebral perfusion pressure (mmHg, mean/SD)	59.2 (8.9)	57.7 (6.3)
Duration mechanical ventilation (days, mean/SD)	11.1 (6.3)	14.8 (7.2)
ICU LoS (days, mean/SD)	30.0 (20.0)	14.3 (6.5)
Hospital LoS (days, mean/SD)	35.8 (21.7)	41.2 (23.2)
ICU mortality (*n*/%)	1 (8.3)	1 (8.3)
Hospital mortality (*n*/%)	1 (8.3)	1 (8.3)
6-month mortality (*n*/%)	2 (16.7)	1 of 9 (11.1)^a^

^a^ Highest ICP recorded in every TIL24 epoch during the 96-h study period for each patient; 6-month mortality missing for three patients in the non-esmolol group.

GCS, Glasgow Coma Score; ICP, intracranial pressure; ICU, intensive care unit; LoS, length of stay; SD, standard deviation; TBI, traumatic brain injury; TIL, Therapy Intensity Level.

**FIG. 1.**
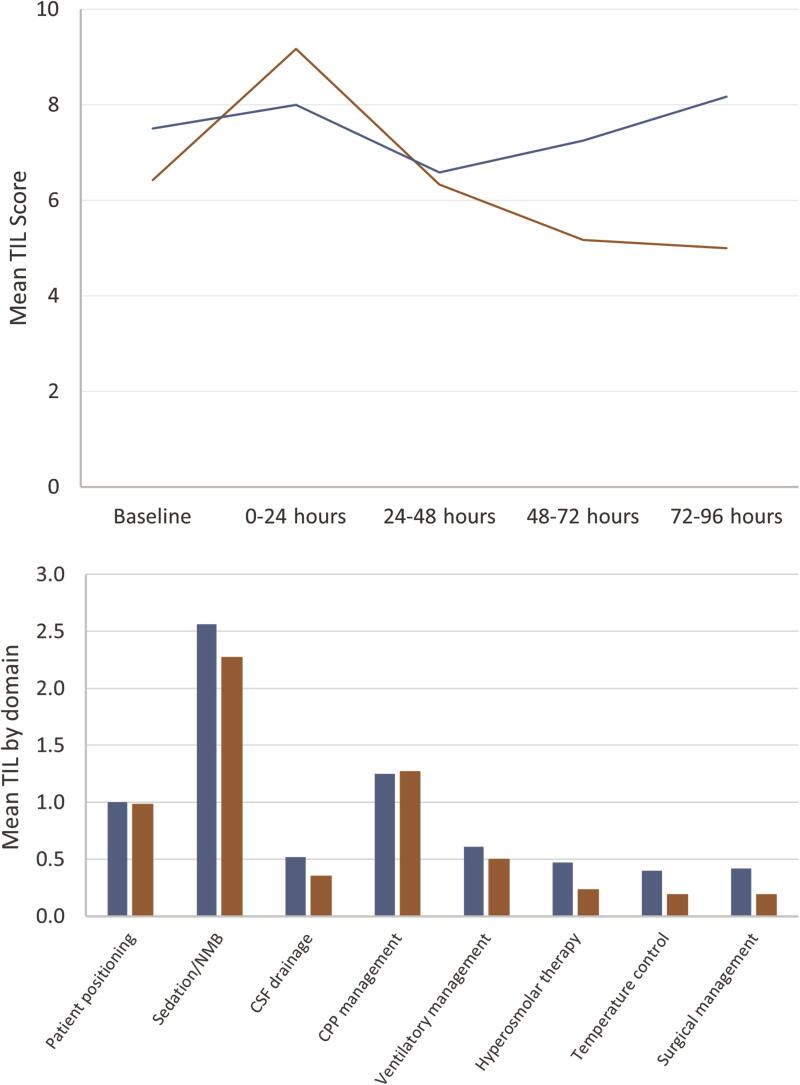
(Top) Therapy Intensity Level (TIL) scores at the baseline and during consecutive 24-h periods for the esmolol (red) and non-esmolol (blue) groups. (Top) Mean scores in each TIL domain over 96 h for the esmolol (red) and non-esmolol (blue) group. CPP, cerebral perfusion pressure; CSF, cerebrospinal fluid.

The TIL24 score in each epoch was (mean/standard deviation) (1) (0–24 h) esmolol 7.5 (5.2) and non-esmolol 7.1 (3.1); (2) (24–48 h) esmolol 5.4 (1.9) and non-esmolol 5.8 (3.7); (3) (48–72 h) esmolol 4.8 (1.5) and non-esmolol 6.6 (5.4); and (4) (72–96 h) esmolol 4.5 (1.5) and non-esmolol 7.0 (4.0), as shown in Figure 1 (top).

The main secondary outcome, mean TIL by domain for each group, is shown in Figure 1 (top). The domain TIL was lower in group E for surgical management, temperature control, hyperosmolar therapy, CSF drainage, sedation, and ventilatory management. There was a slight increase in domain TIL for CPP management in the E group. Other secondary outcomes are shown in Table 1.

## Discussion

In this single-center *post hoc* analysis (of the EBB-TBI study), we have shown that EBB-TBI is associated with a sustained reduction in TIL over the first 4 days after admission. The pattern of reduction differs from that in a contemporaneous cohort not managed with esmolol in whom TIL increases after 48 h in the ICU.

The development of cerebral edema is thought to begin at the time of injury and continue for 2–3 days,^16^ although high-resolution ICP monitoring describes a later peak of ICP pressure-time dose in the slightly later 84–180-h time period.^17^ TIL is a proxy for cerebral edema and subsequent intracranial hypertension because more ICP-lowering interventions are needed as edema progresses.

In the NE group, the timing of TIL24 rebound from 48 h is consistent with this hypothesis and previous observations of the time course of intracranial hypertension. This rebound is not seen in the E group, which suggests EBB might mitigate the development of cerebral edema and intracranial hypertension.

The domain TIL results show that esmolol use does not alter CPP maintenance interventions (fluid or vasopressors for CPP <60 mmHg) but does result in a reduction in need for osmotherapy, CSF drainage, temperature control, and surgical management. Again, this is consistent with a beneficial effect on the development of cerebral edema.

We did not see any compromise of heart rate or CPP. Taken with the similar level of CPP interventions in domain TIL, this suggests esmolol titration to heart rate (as a surrogate for overall sympathetic activation) does not compromise blood pressure and is safe in the setting of a Major Trauma Centre ICU.

This *post hoc* analysis is hypothesis generating and cannot determine the effect on patient-centered outcomes, although we believe there is good reason to continue beta-blocker research on the strength of the results presented. Previous meta-analysis has noted the increase in LoS. Longer hospital LoS in the NE group could reflect slower neurological recovery, in keeping with recent meta-analyses showing improved functional outcome in patients exposed to beta-blockade.^8,9,18^

We are also unable to comment on mechanisms that could determine the effect of esmolol on the extent of cerebral edema. This study does not differentiate between a beta-1 class effect and the direct action of esmolol itself. We would encourage other researchers in the area to include TIL in their outcome measures to assist in answering this question.

The strengths of this study are the protocolized use of esmolol for EBB after severe TBI in adults, selection of a comparable control cohort, and use of TIL, a validated score for this patient group. All patients were otherwise managed according to the same protocol based on widely accepted principles by experienced neurointensivists and neurosurgeons in a Major Trauma Centre.

The limitations are the retrospective nature of data collection, the single-center nature of the study, the potential for bias in the absence of randomization, the lack of information on mechanisms, and the use of a surrogate outcome measure. Although we believe TIL to be a useful and valid outcome measure that appears to be linked to outcome, there is no agreement on what a clinically meaningful reduction in TIL would be. In addition, as TIL could potentially differ depending on the package of interventions used to control ICP, we cannot say our results generalize to all ICUs.

Nevertheless, we believe that the potentially beneficial effect of esmolol on cerebral edema is significant. The absence of direct harm from esmolol use and the additional potential benefit from reduced requirement for hypothermia (known to be harmful) and in other especially surgical interventions make esmolol deserving of further study.^19^

## Conclusion

Esmolol for EBB in adults after severe TBI is associated with reduction in ICP-directed interventions. This study supports further research into mechanisms and effects on patient-centered outcomes of esmolol after TBI.

## Abbreviations Used

APACHE: Acute Physiology and Chronic Health Evaluation
CPP: Cerebral perfusion pressure
CSF: Cerebro-spinal fluid CT Computed tomography
E: Esmolol
EBB: Early beta blockade
GCS: Glasgow Coma Score
h: hours
ICP: Intracranial pressure
IMPACT: International Mission for Prognosis and Analysis of Clinical Trials
ISRCTN: International Standard Randomised Controlled Trial Number
LoS: Length of stay
m: metre
mcg/kg/min: micrograms per kilogram per minute
mmHg: millimetres of mercury
NE: Non-esmolol
SD: Standard deviation
TBI: Traumatic brain injury
TIL: Therapy Intensity Level
